# *PHENIX*: a comprehensive Python-based system for macromolecular structure solution

**DOI:** 10.1107/S0907444909052925

**Published:** 2010-01-22

**Authors:** Paul D. Adams, Pavel V. Afonine, Gábor Bunkóczi, Vincent B. Chen, Ian W. Davis, Nathaniel Echols, Jeffrey J. Headd, Li-Wei Hung, Gary J. Kapral, Ralf W. Grosse-Kunstleve, Airlie J. McCoy, Nigel W. Moriarty, Robert Oeffner, Randy J. Read, David C. Richardson, Jane S. Richardson, Thomas C. Terwilliger, Peter H. Zwart

**Affiliations:** a Lawrence Berkeley National Laboratory, Berkeley, CA 94720, USA; b Department of Bioengineering, UC Berkeley, CA 94720, USA; c Department of Haematology, University of Cambridge, Cambridge Institute for Medical Research, Wellcome Trust/MRC Building, Cambridge CB2 0XY, England; d Department of Biochemistry, Duke University Medical Center, Durham, NC 27710, USA; e Los Alamos National Laboratory, Los Alamos, NM 87545, USA

**Keywords:** *PHENIX*, Python, macromolecular crystallography, algorithms

## Abstract

Macromolecular X-ray crystallography is routinely applied to understand biological processes at a molecular level. How­ever, significant time and effort are still required to solve and complete many of these structures because of the need for manual interpretation of complex numerical data using many software packages and the repeated use of interactive three-dimensional graphics. *PHENIX* has been developed to provide a comprehensive system for macromolecular crystallo­graphic structure solution with an emphasis on the automation of all procedures. This has relied on the development of algorithms that minimize or eliminate subjective input, the development of algorithms that automate procedures that are traditionally performed by hand and, finally, the development of a framework that allows a tight integration between the algorithms.

## Abstract 2

The *PHENIX* software for macromolecular structure determination is described.

## 1. Foundations

### 1.1. *PHENIX* architecture

The *PHENIX* (Adams *et al.*, 2002 ▶) architecture is designed from the ground up as a hybrid system of tightly integrated interpreted (‘scripted’) and compiled software modules. A mix of scripted and compiled components is invariably found in all major successful crystallographic packages, but often the scripting is added as an afterthought in an *ad hoc* fashion using tools that predate the object-oriented programming era. While such *ad hoc* systems are quickly established, they tend to become a severe maintenance burden as they grow. In addition, users are often forced into many time-consuming routine tasks such as manually converting file formats. In *PHENIX*, the scripting layer is the heart of the system. With only a few exceptions, all major functionality is implemented as modules that are exclusively accessed *via* the scripting interfaces. The object-oriented Python scripting language (Lutz & Ascher, 1999 ▶) is used for this purpose. In about two decades, a large developer/user community has produced millions of lines of highly uniform, interoperable, mature and openly available sources covering all aspects of programming ranging from simple file handling to highly sophisticated network communication and fully featured cross-platform graphical interfaces. Embedding crystallographic methods into this environment enables an unprecedented degree of automation, stability and portability. By design, the object-oriented programming model fosters shared collaborative development by multiple groups. It is routine practice to hierarchically recombine modules written by different groups into ever more complex procedures that appear uniform from the outside. A more detailed overview of the key software technology leading to all these advances, presented in the context of crystallography, can be found in Grosse-Kunstleve *et al.* (2002 ▶).

In addition to the advantages outlined in the previous paragraph, the scripting language is generally most efficient for the rapid development of new algorithms. However, run­time performance considerations often dictate that numerically intensive calculations are eventually implemented in a compiled language. The first choice of a compiled language is of course to reuse the same language environment as used for the scripting language itself, which is a C/C++ environment. Not only is this the mainstream software environment on all major platforms used today, but with probably hundreds of millions of lines of C/C++ sources in existence it is an environment that is virtually guaranteed to thrive in the long term. An in-depth discussion of the combined use of Python and C++ can be found in Grosse-Kunstleve *et al.* (2002 ▶) and Abrahams & Grosse-Kunstleve (2003 ▶). This model is used throughout the *PHENIX* system.

### 1.2. Graphical user interface

A new graphical user interface (GUI) for *PHENIX* was introduced in version 1.4. It uses the open-source wxPython toolkit, which provides a ‘native’ look on each operating system. Development has focused on providing interfaces around the existing command-line programs with minimal modification, using the same underlying configuration system (*libtbx.phil*) as used by most *PHENIX* programs as a template to automatically generate controls. Because these programs are implemented primarily as Python modules, complex data including models, reflections and other viewable data may be exchanged with the GUI without resorting to parsing log files. The current *PHENIX* release (version 1.5) includes GUIs for *phenix.refine* (Afonine *et al.*, 2005 ▶), *phenix.xtriage* (Zwart *et al.*, 2005 ▶), the *AutoSol* (Terwilliger *et al.*, 2009 ▶), *AutoBuild* (Terwilliger, Grosse-Kunstleve, Afonine, Moriarty, Adams *et al.*, 2008 ▶) and *LigandFit* (Terwilliger *et al.*, 2006 ▶) wizards, the restraints editor *REEL*, all of the validation tools and several utilities for creating and manipulating maps and reflection files. More recent builds of *PHENIX* contain a new GUI for the *AutoMR* wizard and future releases will include a new interface for *Phaser* (McCoy *et al.*, 2007 ▶).

Intrinsically graphical data is visualized with embedded graphs (using the free matplotlib Python library) or a simple OpenGL viewer. This simplifies the most complex parameters, such as atom selections in *phenix.refine*, which can be visual­ized or picked interactively with the built-in viewer. The GUI also serves as a platform for additional automation and user customization. Similarly to the *CCP*4 interface (*CCP*4*i*; Potterton *et al.*, 2003 ▶), *PHENIX* manages data and task history for separate user-defined projects. Default parameters and input files can be specified for each project; for instance, the generation of ligand restraints from the *phenix.refine* GUI gives the user the option of automatically loading these restraints in future runs.

The popularity of Python as a scientific programming language has led to its use in many other structural-biology applications, especially molecular-graphics software. The *PHENIX* GUI includes extension modules for the modeling programs *Coot* (Emsley & Cowtan, 2004 ▶) and *PyMOL* (DeLano, 2002 ▶), both of which are controlled remotely from *PHENIX* using the XML-RPC protocol. This allows the interfaces to integrate seamlessly; any model or map in *PHENIX* can be automatically opened in *Coot* with a single click. In programs that iteratively rebuild or refine structures, such as *AutoBuild* and *phenix.refine*, the current model and maps will be continually updated in *Coot* and/or *PyMOL* as soon as they are available. In the validation utilities, clicking on any atom or residue flagged for poor statistics will recentre the graphics windows on that atom. Remote control of the *PHENIX* GUI is also simple using the same protocol and simple extensions to the *Coot* interface provide direct launching of *phenix.refine* with a model pre-loaded.

## 2. Analysis of experimental data

*PHENIX* has a range of tools for the analysis, validation and manipulation of X-ray diffraction data. A comprehensive tool for analyzing X-ray diffraction data is *phenix.xtriage* (Zwart *et al.*, 2005 ▶), which carries out tests ranging from space-group determination and detection of twinning to detection of anomalous signal. These tests provide the user and the various wizards with a set of statistics that characterize a data set. For analysis of twinning, *phenix.xtriage* consolidates a number of statistics to provide a balanced verdict of possible symmetry and twin-related issues with the data. *Phenix.xtriage* provides the user with feedback on the overall characteristics of the data. Routine usage of *phenix.xtriage* during or immediately after data collection has resulted in the timely discovery of twinning or other issues (Flynn *et al.*, 2007 ▶; Kostelecky *et al.*, 2009 ▶). Detection of these idiosyncrasies in the data typically reduces the overall effort in a successful structure determination.

A likelihood-based estimation of the overall anisotropic scale factor is performed using the likelihood formalism described by Popov & Bourenkov (2003 ▶). Database-derived standard Wilson plots for proteins and nucleic acids are used to detect anomalies in the mean intensity. These anomalies may arise from ice rings or other issues (Morris *et al.*, 2004 ▶). Data strength and low-resolution completeness are also analysed. The presence of anomalous signal is detected by analysis of the measurability, a quantity expressing the fraction of statistically significant Bijvoet differences in a data set (Zwart, 2005 ▶). The native Patterson function is used to detect the presence of pseudo-translational symmetry. A database-derived empirical distribution of maximum peak heights is used to assign significance to detected peaks in the Patterson function.

A comprehensive automated twinning analysis is per­formed. Twin laws are derived from first principles to facilitate the identification of pseudo-merodehral cases. Amplitude and intensity ratios, 〈|*E* ^2^ − 1|〉 values, the *L*-statistic (Padilla & Yeates, 2003 ▶) and *N*(*Z*) plots are derived from data cut to the resolution limit suggested by the data-strength analysis. The removal of shells of data with relatively high noise content greatly improves the automated interpretation of these statistics. A Britton plot, *H*-test and a likelihood-derived approach are used to estimate twin fractions when twin laws are present. If a model has been supplied, an *R versus R* (Lebedev *et al.*, 2006 ▶) analysis is carried out. This type of analysis is of particular use when dealing with pseudo-symmetry, space-group problems and twinning (Zwart *et al.*, 2008 ▶).

To test for inconsistent indexing between different data sets, a set of reindexing laws is derived from first principles given the unit cells and space groups of the sample and reference data sets. A correlation analysis suggests the most likely choice of reindexing of the data. Analysis of the metric symmetry of the unit cell provides a number of likely point groups. A likelihood-inspired method is used to suggest the most likely point group of the data. Subsequent analysis of systematic absences in a likelihood framework ranks subsequent space-group possibilities (details to be published).

## 3. Substructure determination, phasing and molecular replacement

After ensuring that the diffraction data are sound and understood, the next critical necessity for solving a structure is the determination of phases using one of several strategies (Adams, Afonine *et al.*, 2009 ▶).

### 3.1. Substructure determination

The substructure-determination procedure implemented as *phenix.hyss* (*Hybrid Substructure Search*; Grosse-Kunstleve & Adams, 2003 ▶) combines the multi-trial dual-space recycling approaches pioneered by *Shake-and-Bake* (Miller *et al.*, 1994 ▶) and later *SHELXD* (Sheldrick, 2008 ▶) with the use of the fast translation function (Navaza & Vernoslova, 1995 ▶; Grosse-Kunstleve & Brunger, 1999 ▶). The fast translation function is the basis for a systematic search in the Patterson function (performed in reciprocal space), in contrast to the stochastic alternative of *SHELXD* (performed in direct space). *Phenix.hyss* is the only substructure-determination program to fully integrate automatic comparison of the substructures found in multiple trials *via* a Euclidean Model Matching procedure (part of the *cctbx* open-source libraries). This allows *phenix.hyss* to detect if the same solution was found multiple times and to terminate automatically if this is the case. Extensive tests with a variety of SAD data sets (Grosse-Kunstleve & Adams, 2003 ▶) have led to a parameterization of the procedure that balances runtime considerations and the likelihood that repeated solutions present the correct substructure. In many cases the procedure finishes in seconds if the substructure is detectable from the input data.

### 3.2. Phasing

*Phaser*, available in *PHENIX* as *phenix.phaser*, applies the principle of maximum likelihood to solving crystal structures by molecular replacement, by single-wavelength anomalous diffraction (SAD) or by a combination of both. The likelihood targets take proper account of the effects of different sources of error (and, in the case of SAD phasing, their correlations) and allow different sources of information to be combined. In solving a molecular-replacement problem with a number of different components, the information gained from a partial solution increases the signal in the search for subsequent components. Because the likelihood scores for different models can be directly compared, decisions among models can readily be made as part of automation strategies (discussed below).

### 3.3. Noncrystallographic symmetry (NCS)

Noncrystallographic symmetry is an important feature of many macromolecular crystals that can be used to greatly improve electron-density maps. *PHENIX* has tools for the identification of NCS and for using NCS and multiple crystal forms of a macromolecule in phase improvement.

*Phenix.find_ncs* and *phenix.simple_ncs_from_pdb* are tools for the identification of noncrystallographic symmetry in a structure using information from a heavy-atom substructure or an atomic model. *Phenix.simple_ncs_from_pdb* will identify NCS and generate transformations from the chains in a model in a PDB file. *Phenix.find_ncs* will identify NCS from either a heavy-atom substructure (Terwilliger, 2002*a* ▶) or the chains in a PDB file and will then compare this NCS with the density in a map to verify that the NCS is actually present.

*Phenix.multi_crystal_average* is a method for combining information from several crystal forms of a structure. It is especially well suited to cases where each crystal form has its own NCS, adjusting phases for each crystal form so that all the NCS copies in all crystals are as similar as possible.

NCS restraints should normally be applied in density modification and model building in all cases except where there is clear evidence that NCS is not present. In density modification within *PHENIX* the presence of NCS is identified from the heavy-atom sites or from an atomic model if available. The local correlation of density in NCS-related locations is then used automatically to set variable restraints on NCS symmetry in the map. In refinement, NCS symmetry is applied through coordinate restraints, targeting the positions of each NCS copy relative to those of the other NCS-related chains. The default NCS restraints in *PHENIX* are very tight, with targets of 0.05 Å r.m.s. At resolutions lower than about 2.5 Å these tight restraints on NCS should usually be applied. At higher resolutions it may be appropriate to use looser restraints or to remove them altogether. Additionally, if there are segments of the chains that clearly do not obey the NCS relationships they should be excluded from the NCS restraints. Normally this is performed automatically, but it can also be specified explicitly.

## 4. Model building, ligand fitting and nucleic acids

Key steps in the analysis of a macromolecular crystal structure are building an initial core model, identification and fitting of ligands into the electron-density map and building an atomic model for loop regions that are less well defined than the majority of the structure. *PHENIX* has tools for rapid model building of secondary structure and main-chain tracing (*phenix.find_helices_strands*) and for the fitting of flexible ligands (*phenix.ligandfit*) as well as for fitting a set of ligands to a map (*phenix.find_all_ligands*) and for the identification of ligands in a map (*phenix.ligand_identification*). *PHENIX* additionally has a tool for the fitting of missing loops (*phenix.fit_loops*). Validation tools are provided so that the models produced can be validated at each step along the way.

### 4.1. Model building

*Phenix.find_helices_strands* will rapidly build a secondary-structure-only model into a map or very rapidly trace the polypeptide backbone of a model into a map. To build secondary structure in a map, *phenix.find_helices_strands* identifies α-helical regions and β-strand segments, models idealized helices and strands into the corresponding density, allowing for bending of the helices and strands, and assembles these into a composite model. To very rapidly trace the main chain in a map,* phenix.find_helices_strands* finds points along ridgelines of high density where C_α_ atoms might be located, identifies pairs and then triplets of these C_α_ atoms that have density between the atoms and plausible geometry, constructs all possible connections of these C_α_ atoms into nonamers and then identifies all the longest possible chains that can be made by joining the nonamers. This process can build a C_α_ model at a rate of about 20 residues per second, yielding a backbone model that can readily be interpreted visually or automatically to evaluate the quality of the map that it is based on.

*Phenix.fit_loops* will fit missing loops in an atomic model. It uses *RESOLVE* model building (Terwilliger, 2003*a* ▶,*b* ▶,*c* ▶) to extend the chain from either end where a loop is missing and to connect the chains into a loop with the expected number of residues.

### 4.2. Ligand fitting

*Phenix.ligandfit* is a tool for fitting a flexible ligand into an electron-density map (Terwilliger *et al.*, 2006 ▶). The key approaches used are breaking the ligand into its component rigid-body parts, finding where each of these can be placed into density, tracing the remainder of the ligand based on the positions of these core rigid-body parts and recombining the best parts of multiple fits while scoring based on the fit to the density.

*Phenix.find_all_ligands* is a tool for finding all the instances of each of several ligands in an electron-density map. *Phenix.find_all_ligands* finds the largest contiguous region of unused density in a map and uses *phenix.ligandfit* to fit each supplied ligand into that density. It then chooses the ligand that has the highest real-space correlation to the density (Terwilliger, Adams *et al.*, 2007 ▶). It then repeats this process until no ligands can be satisfactorily fitted into any remaining density in the map.

*Phenix.ligand_identification* is a tool for identifying which ligands are compatible with unknown electron density in a map (Terwilliger, Adams *et al.*, 2007 ▶). It can search using the 200 most common ligands from the PDB or from a user-supplied list of ligands. *Phenix.ligand_identification* uses *phenix.ligandfit* to fit each ligand to the map and identifies the best-fitting ligand using the real-space correlation and surface complementarity of the ligand and the atoms in the structure surrounding the ligand-binding site.

### 4.3. RNA and DNA

In common with most macromolecular crystallographic tools, *PHENIX* was originally developed with protein structures primarily in mind. Now that nucleic acids, and especially RNA, are increasingly important in large biological structures, the system is being modified in places where subtle differences in procedure are needed rather than just the relevant libraries.

Model building in *phenix.autobuild* now has a preliminary set of nucleic acid procedures that take advantage of the relatively well determined phosphate and base positions, as well as the preponderance of double helix, and that make use of the RNA backbone conformers recently defined by the RNA Ontology Consortium (Richardson *et al.*, 2008 ▶). Nucleic acid structures benefit significantly from torsion-angle refinement, which has recently been added to the options in *phenix.refine*. A principal problem in RNA models is getting the ribose pucker correct, although it is known to consist almost entirely of either C3′-*endo* (which is commoner and that found in the A-form helix) or C2′-*endo* (Altona & Sundaralingam, 1972 ▶). *MolProbity* uses the perpendicular distance from the 3′ phosphate to the line of the C1′—N1/9 glycosidic bond as a reliable diagnostic of ribose pucker (Davis *et al.*, 2007 ▶; Chen *et al.*, 2010 ▶). This same test has now been built into *phenix.refine* to allow the use of pucker-specific target parameters for bond lengths, angles and torsions (Gelbin *et al.*, 1996 ▶) rather than the uneasy compromise values (Parkinson *et al.*, 1996 ▶) used in most pucker-agnostic refinement. Currently, if an incorrect pucker is diagnosed it must usually be fixed by user rebuilding, for instance in *Coot* (Emsley & Cowtan, 2004 ▶) or in *RNABC* (Wang *et al.*, 2008 ▶). A rebuilding functionality will probably be incorporated into *PHENIX* soon, but in the meantime the refinement will now correctly maintain the geometry of a C2′-*­endo* pucker once it has been built and identified using conformation-specific residue names.

### 4.4. Maps, models and avoiding bias

*Phenix.refine* (and the graphical tool *phenix.create_maps*) can produce various types of maps, including anomalous difference, maximum-likelihood weighted (p*mF_obs_ − q*DF_model_)exp(*i*α_model_) and regular (p*F_obs_ − q*F_model_)exp(*i*α_model_), where *p* and *q* are any user-defined numbers, filled and kick maps. The coefficients *m* and *D* of likelihood-weighted maps (Read, 1986 ▶) are computed using test-set reflections as described in Lunin & Skovoroda (1995 ▶) and Urzhumtsev *et al.* (1996 ▶).

Data incompleteness, especially systematic incompleteness, can cause map distortions (Lunin, 1988 ▶; Tronrud, 1997 ▶). An approach to remedying this problem is to replace (‘fill’) missing observations with nonzero values. One can use *DF*_model_ (similarly to *REFMAC*; Murshudov *et al.*, 1997 ▶) to replace the missing *F*_obs_ or use 〈*F*_obs_〉, where the *F*_obs_ are averaged across a resolution bin around the missing *F*_obs_ value. Based on a limited number of tests, both ‘filling’ schemes produce similar results, reiterating the importance of phases. However, it is important to keep in mind that by replacing missing *F*_obs_ there is a risk of introducing bias and obviously the more incomplete the data is the larger the risk. At present it is advisable to use both maps simultaneously: filled and not filled.

An average kick map (AK map; Gunčar *et al.*, 2000 ▶; Turk, 2007 ▶; Pražnikar *et al.*, 2009 ▶) is the result of the following procedure. A large ensemble of structures is created where the coordinates of each structure from the ensemble are all randomly shaken. A map is then computed for each structure. Finally, all maps are averaged to generate one AK map. An AK map is expected to have less bias and less noise and to enhance the existing signal and can potentially clarify some initially bad densities.

A computationally intensive but powerful method of creating a very low-bias map is to carry out iterative model building and refinement while omitting one region of the map from all calculations of structure factors (Terwilliger, Grosse-Kunstleve, Afonine, Moriarty, Adams *et al.*, 2008 ▶). The *phenix.autobuild* iterative-build OMIT map procedure carries this out automatically for either a single OMIT region or for overlapping OMIT regions to create a composite iterative-build OMIT map.

## 5. Model, and model-to-data, validation

The result of crystallographic structure determination is the atomic model. There are three principal components in assessing model quality: the covalent model geometry, the model stereochemistry and the quality of fit between the model and experimental data in both real space and in reciprocal space. All three provide overall measures, and the first two plus the real-space aspect of the third also provide checks for local outliers, which give the best leverage for user intervention to actively improve model accuracy (Arendall *et al.*, 2005 ▶). (Validation of the experimental data was described in §[Sec sec2]2 above.) *PHENIX* includes many individual tools for specific aspects of validation, plus several systems that combine those results into overall summaries. Validation is provided both for user evaluation of the progress and results of a structure solution and also to help inform the automated choices made by other parts of the system.

Most aspects of the *MolProbity* model-validation tools (Davis *et al.*, 2007 ▶; Chen *et al.*, 2010 ▶) have been adapted or rewritten for integrated use within *PHENIX* and are pre­sented to the user by the new GUI (§[Sec sec1.2]1.2). H atoms are added by *phenix.reduce*, with optimization of entire local hydrogen-bond networks, consideration of the first layer of crystallo­graphic waters and optional correction of side-chain amide or histidine 180° ‘flips’ (Word, Lovell, Richardson *et al.*, 1999 ▶). All-atom contacts (Word, Lovell, LaBean *et al.*, 1999 ▶) are calculated by *phenix.probe*, which provides the atomic overlap information needed for the validation of serious all-atom steric clashes and can also be visualized in *Coot*. For the *PHENIX* GUI, the set of *MolProbity*-based tools provides both overall model statistics, such as clashscore and percentage of outliers, and detailed lists of the Ramachandran (Lovell *et al.*, 2003 ▶), rotamer (Lovell *et al.*, 2000 ▶), C_β_ deviation (Lovell *et al.*, 2003 ▶) and clash outliers. Command-line tools are available for these validation methods: *phenix.rotalyze*, *phenix.ramalyze*, *phenix.cbetadev*, *phenix.clashscore*, *phenix.reduce* and *phenix.probe*. Additionally, *phenix.validate_model*, which analyzes the deviations of bond lengths, bond angles, planarity *etc*. from ideal library values, complements the *MolProbity* torsional and atomic clash tools.

*Phenix.real_space_correlation* asserts the local model-to-data correspondence by providing a quantitative measure of how the atomic model fits the electron-density map at the residue or atom level (depending on the resolution). Rapidly obtaining a snapshot of global figures of merit for a crystallo­graphic model and associated experimental data is a frequent task that is performed at all stages of structure solution. This task can be complicated for several reasons: the presence of novel ligands or nonstandard residues in the PDB-format (Berman *et al.*, 2000 ▶) coordinate file, data collected from twinned crystals, various reflection datafile formats, different representation of atomic displacement parameters in the presence of TLS (Schomaker & Trueblood, 1968 ▶), experimental data type (X-­ray and/or neutron), files with multiple models and various formatting issues. *Phenix.model_vs_data* is designed to automatically handle all these complications with minimal user input (a PDB file and a reflection data file) and provide a concise summary output. *Phenix.polygon* (Urzhumtseva *et al.*, 2009 ▶) is a graphical tool that is designed to indicate the similarity of validation parameters, such as free *R* value, for a particular structure compared with those deposited in the PDB. This comparison is performed for all other structures solved at similar resolution limits. The result is presented graphically. *Phenix.validation* combines all of the tools described above in one GUI, providing a single place for assessing the results of structure determination.

### 5.1. Model and structure-factor manipulation and analysis

*PHENIX* has a range of tools for displaying, analyzing and manipulating structure-factor and model information. *Phenix.mtz.dump* and *phenix.cif_as_mtz* display and convert structure-factor data. *Phenix.print_sequence*, *phenix.pdb_atom_selection* and *phenix.pdbtools* display and manipulate coordinate files.

*Phenix.tls* is a tool for the extraction and manipulation of TLS information. Using this tool, TLS matrices and selections can be extracted from *REFMAC*- or *PHENIX*-formatted PDB file headers and the total or residual atomic *B* factors can be computed and output. Future functionality will include the complete analysis of TLS matrices and their graphical visual­ization.

*Phenix.get_cc_mtz_mtz* and *phenix.get_cc_mtz_pdb* are tools for analyzing the agreement between maps based on a pair of MTZ files or between maps calculated from an MTZ file and a PDB file. The key attributes of these tools are that they automatically search all allowed origin shifts that might relate the two maps and that they write out a modified version of one of the MTZ files or of the PDB file, shifted to match the other.

## 6. Structure refinement

*Phenix.refine* is the state-of-the-art crystallographic structure-refinement engine of *PHENIX*. The foundational refinement machinery is a combination of highly efficient programming tools and new or rethought crystallographic algorithms. *Phenix.refine* possesses an extensive set of tools that cover the majority of refinement scenarios at any data resolution from low to ultrahigh. Various reflection-data formats (for example, *CNS*, MTZ and *SHELX*) are recognized automatically. The input experimental data are checked for outliers (Read, 1999 ▶; Zwart *et al.*, 2005 ▶) and any reflections identified as such are excluded from the refinement calculations. Twinning can also be taken into account by providing a twin-law operator, which can be obtained using *phenix.xtriage*. Both X-ray and/or neutron diffraction data can be used and an option for joint XN refinement is available (simultaneous refinement against X-­ray and neutron data; Adams, Mustyakimov *et al.*, 2009 ▶). Each refinement run begins with robust mask-based bulk-solvent correction and anisotropic scaling (Afonine *et al.*, 2005 ▶). Tools such as efficient rigid-body refinement (multiple-zones algorithm; Afonine *et al.*, 2009 ▶), simulated-annealing refinement (Brünger *et al.*, 1987 ▶) in Cartesian or torsion-angle space (Grosse-Kunstleve *et al.*, 2009 ▶), automatic NCS detection and its use as restraints in refinement are important at low resolution and in the initial stages of refinement. A broad range of atomic displacement parameterizations are available, including grouped isotropic, constrained anisotropic (TLS) and individual atomic isotropic or anisotropic, allowing efficient modelling of atomic displacement parameters at any resolution. Occupancy refinement (grouped, individual, group constrained for alternative conformations or any mixture) can be performed for any user-defined atoms. Atoms in alternative conformations are recognized automatically based on altLoc identifiers in the input PDB file and their occupancies are refined by default. Ordered solvent (water) model updating is integrated into the refinement process.

The availability of ultrahigh-resolution data makes it possible to visualize the residual density arising from bonding effects; *phenix.refine* employs a novel interatomic scatterers model (Afonine *et al.*, 2007 ▶) to adequately account for these features. A flexible parameterization of H atoms allows their use at any resolution from subatomic (where their parameters can be refined individually) to low resolution (where a riding model is used). Refinement can be performed using a variety of refinement target functions, including maximum likelihood, maximum likelihood with experimental phase information and amplitude least squares. The refinement of coordinates can be performed in real or reciprocal space (allowing dual-space refinement). Novel ligands can easily be included in refinement by providing a corresponding CIF file as input (the CIF file can be automatically created using *phenix.ready_set*).

Manual fixing of amino-acid side-chain rotamers can be time-consuming, especially for large structures. Although the use of simulated-annealing refinement increases the convergence radius, it can still fail to fit incorrectly modelled side chains into the correct density. *Phenix.refine* has an option for automatic selection of the best rotamer based on a rotamer library (Lovell *et al.*, 2000 ▶) and optimal fit into the density (details to be published elsewhere). Furthermore, coupling real-space refinement with the built-in rotamer library and available *MolProbity* tools allows the automated identification and robust correction of common systematic errors involving backward-fit conformations for Leu, Thr, Val, Ile and Arg side chains, as developed and tested in the *Autofix* method (Headd *et al.*, 2009 ▶).

*Phenix.refine* allows multi-step complex refinement protocols in which most of the available refinement strategies can be combined with each other and applied to any selected part of the model. For example, a run of *phenix.refine* may perform rigid-body refinement, simulated annealing, individual and grouped *B* factors combined with TLS refinement, constrained occupancy refinement and automatic water picking.

The output of *phenix.refine* includes various maps (maximum-likelihood weighted, kicked, incompleteness corrected, anomalous difference and those with any user-defined coefficients), complete model and data statistics and PDB file with a formatted REMARK 3 header ready for PDB deposition. The *phenix.refine* GUI is integrated with *Coot* and *PyMOL*, allowing seamless visual analysis of the refined model and associated maps.

*Phenix.refine* is tightly integrated with other *PHENIX* components, making structure solution, building and refinement a one-step process (for example, in the *AutoMR* and *AutoBuild* wizards). It is routinely tested by automatic re-refinement of all models in the PDB for which the experimental data are available.

### 6.1. Ligand-coordinate and restraint-geometry generation

The *electronic Ligand Builder and Optimization Builder* (*eLBOW*; Moriarty *et al.*, 2009 ▶) is a suite of tools designed for the reliable generation of Cartesian coordinates and geometry restraints for both novel and known ligands. In line with the rest of the *PHENIX* package, the *eLBOW* modules are written in Python, with the numerically intensive portions of the code written in C++. *eLBOW* is a flexible platform for converting a majority of common chemical inputs to optimized three-dimensional coordinates and geometry restraints for refinement. Ligand geometries can be minimized using the semi-empirical AM1 quantum-chemical method (Stewart, 2004 ▶), a numerically efficient and chemically accurate technique for the class of molecules commonly complexed with or bound to proteins.

In addition, a graphical user interface for editing geometry restraints and simple geometry manipulation of ligands has been developed. The *Restraints Editor, Especially Ligands* (*REEL*) removes the tedium of manually editing a restraints file by providing a number of commonly performed actions *via* pull-down menus and other interactive features. The effect of changes in the restraints can be immediately reflected in the molecule view to provide user feedback.

A tool that uses many of the features of *eLBOW* to quickly and easier prepare a protein model for refinement is known as *ReadySet!* The flexibility of the Python interface is exemplified by the use of *Reduce*, *eLBOW* and several smaller portions of the *cctbx* toolkit to add H and/or D atoms to the model, ligands and water and to generate metal-coordination files and geometry restraints for unknown ligands. The files required for covalently bound ligands are also generated.

## 7. Integrated structure determination

### 7.1. Why automation?

Automation has dramatically changed macromolecular crystallography over the past decade, both by greatly speeding up the process of structure solution, model building and refinement and by bringing the tools for structure determination to a much wider group of scientists. As automation becomes increasingly comprehensive, it will allow users to test many more possibilities for structure determination, will allow improved estimation of uncertainties in the final structures and will allow the determination of ever more complex and difficult structures.

The *PHENIX* environment has been developed with automation as a key and defining feature. Each tool within *PHENIX* can seamlessly and nearly effortlessly be incorporated as part of any other tool or process in *PHENIX*. This means that very complex tasks can be built up from well tested and characterized tools and that tools and higher-level methods can be re-used in many different contexts. With a full automatic regression testing system as an integral part of the *PHENIX* environment, all these tasks and high-level methods are tested daily to ensure the integrity of the entire *PHENIX* system.

### 7.2. Automated structure solution

*PHENIX* has fully integrated structure-solution capability for both experimental phasing (MAD, SAD, MIR and com­binations of these), carried out by *phenix.autosol*, and for molecular replacement, performed by *phenix.automr*. Each of these automated procedures feeds directly into the iterative model building, density modification and refinement of *phenix.autobuild*.

*Phenix.autosol* is designed to allow complete automation of experimental phasing while allowing a high degree of flexibility for advanced users. Beginning with structure-factor amplitudes and the sequence of the macromolecule, *phenix.autosol* uses *phenix.solve* (Terwilliger & Berendzen, 1999 ▶) to scale all data sets, *phenix.xtriage* (Zwart *et al.*, 2005 ▶) to analyze the data for twinning and to correct any anisotropy in the data and *phenix.hyss* (Grosse-Kunstleve & Adams, 2003 ▶) to find potential heavy-atom or anomalously scattering atoms. *Phenix.autosol* carries out experimental phasing with *phenix.phaser* (McCoy *et al.*, 2004 ▶, 2007 ▶) or *phenix.solve* (Terwilliger & Berendzen, 1999 ▶), density modification with *phenix.resolve* (Terwilliger, 1999 ▶) and preliminary model building using the methods in *phenix.autobuild* (Terwilliger, Grosse-Kunstleve, Afonine, Moriarty, Zwart *et al.*, 2008 ▶).

A key step in automated structure solution is the identification of which of several possible space-group and heavy-atom or anomalously scattering-atom substructures is correct. *Phenix.autosol* uses a Bayesian scoring algorithm based on analysis of the experimental electron-density maps to identify which substructures lead to the best maps (Terwilliger *et al.*, 2009 ▶). The main features of the maps that are used in this evaluation are the skewness of the electron density (non-Gaussian histogram of density with more density in the positive tail than the negative tail) and the correlation of local r.m.s. density (large contiguous regions of high variation where the molecule is located and separate large contiguous regions of low variation where the solvent is located).

*Phenix.autosol* is highly flexible, allowing any combination of experimental data, such as MAD + SIRAS or several SAD data sets. Although it is fully automated, the user can control nearly all aspects of the operation of the procedure, including the scoring criteria and decisions about how certain *phenix.autosol* should be that the correct solution is contained in the current lists of solutions.

*Phenix.autosol* can carry out phasing using a combination of experimental SAD data and molecular-replacement information. If a molecular-replacement model is available, *phenix.autosol* will use *phenix.phaser* (McCoy *et al.*, 2004 ▶, 2007 ▶) to complete the anomalous substructure iteratively by con­structing log-likelihood gradient maps for the anomalous scatterers based on the model of the non-anomalous structure and any anomalous scatterers that have already been found. The anomalous substructure is then used along with the model to calculate phases with *phenix.phaser*.

*Phenix.automr* carries out automated likelihood-based molecular replacement using *phenix.phaser* (Read, 2001 ▶; McCoy *et al.*, 2005 ▶, 2007 ▶; McCoy, 2007 ▶). The procedure is highly automated, allowing several copies of each of several components to be placed in a single run, which can also test different possible choices of space group. If there are alternative choices of model for a component, the molecular-replacement calculation can try each of them in turn or combine them as a statistically weighted ensemble. Although the evaluation of the likelihood targets is slow (Read, 2001 ▶), the use of fast approximations for the rotation search (Storoni *et al.*, 2004 ▶) and the translation search (McCoy *et al.*, 2005 ▶) gives run times that are competitive with traditional Patterson-based methods. Likelihood has been demonstrated to be more sensitive to the correct solution, particularly in difficult cases (Read, 2001 ▶). When there are several copies or several components to place, the ability of the likelihood functions to take advantage of preliminary partial solutions can provide a crucial increase in the signal.

### 7.3. Iterative model building, density modification and refinement

*Phenix.autobuild* is a highly integrated and automated procedure for model building and model improvement through iterative model building, density modification and refinement. *Phenix.autobuild* uses *phenix.resolve* (Terwilliger, 2003*a* ▶,*b* ▶) to carry out model building, model extension, model assembly, loop fitting and building outside existing models. It further uses *phenix.resolve* to improve electron-density maps with statistical density modification, including information from the newly built models as well as that obtained from experiment (*e.g. phenix.autosol*), from NCS (Terwilliger, 2002*b* ▶) and from other expected features of electron-density maps such as a flat solvent (Wang, 1985 ▶), the presence of secondary-structural features (Terwilliger, 2001 ▶) and the presence of local patterns of density characteristic of macromolecules (Terwilliger, 2003*c* ▶). To reduce model bias in the procedure, prime-and-switch phasing can also be used (Terwilliger, 2004 ▶). *Phenix.autobuild* uses *phenix.refine* (Afonine *et al.*, 2005 ▶) throughout this process to improve the quality of the models that are built.

*Phenix.autobuild* provides two complementary approaches to model building. For cases in which no model or only a preliminary model has been built, *phenix.autobuild* will con­struct a new model considering the main chain of any supplied models as potential coordinates. In cases where a nearly final model is available, *phenix.autobuild* can apply a rebuild-in-place approach in which the polypeptide chain is rebuilt a few residues at a time without changing the register or the overall features of the model.

The rebuild-in-place approach in *phenix.autobuild* provides a powerful method for the assessment of uncertainties in an atomic model by repetitive rebuilding of the model using different random seeds for each iteration (Terwilliger, Grosse-Kunstleve *et al.*, 2007 ▶). The variability in the coordinates of each atom in the ensemble that is created is a lower bound on the uncertainty of the position of that atom.

## 8. Conclusions

Advances in computational methods and algorithms have made it possible to automate the solution of many structures with *PHENIX*. However, many challenges still exist. In particular, the development of automated methods that can be applied at low resolution (worse than 3.0 Å) remains a priority. In this resolution range there are typically too few experimental data to uniquely define the macromolecular structure for automated *ab initio* model building. Thus, methods are required that rely on prior knowledge from existing macromolecular structures to permit productive automated data interpretation. These methods will need to be developed and applied for all stages of structure solution and tightly integrated to maximize the information extracted from the experimental data.
